# The relationship between attitude and self-efficacy of labor support with supportive behaviors from the perspective of midwives

**DOI:** 10.1186/s12912-023-01197-w

**Published:** 2023-02-07

**Authors:** Khadijeh Heidari, Leila Amiri-Farahani, Sally Pezaro

**Affiliations:** 1 Department of Reproductive Health and Midwifery, School of Nursing and Midwifery, Iran University of Medical Sciences, Tehran, Iran; 2 Department of Reproductive Health and Midwifery, Nursing and Midwifery Care Research Center (NCRC), School of Nursing and Midwifery, Iran University of Medical Sciences, Tehran, 1996713883 Iran; 3 The University of Notre Dame, Australia and Assistant Professor, The Centre for Healthcare Research, Coventry University, Coventry, UK

**Keywords:** Attitude, Self-efficacy, Supportive labor behavior, Labour, Midwife

## Abstract

**Background and aim:**

Effective support given by a midwife during labor and childbirth is associated with numerous positive outcomes. Yet the delivery of such support can be hindered by negative workplace cultures. The purpose of the current study was to examine the relationship between attitude and self-efficacy of labor support and labor supportive behaviors from the perspectives of midwives working in Iran.

**Methods:**

Midwives (*n* = 213) employed in the labor wards of selected hospitals in an urban area of Iran participated in this cross-sectional study. Participants were recruited via convenience sampling from December 2016 to September 2017. The data were collected using a personal characteristics tool, the Labor Support Questionnaire, the Self-efficacy Labor Support Scale, and attitudes toward the Labor Support Questionnaire. Descriptive statistics along with multiple linear regression was used for data analysis.

**Results:**

Participants had a supportive behavior score of 74.98 for mean (SD ± 13.39). The informational support dimension had the highest reported score of the supportive behaviors, whereas the tangible support dimension had the lowest score. The mean scores of attitude and self-efficacy toward labor support were 24.79 (SD ± 4.14) and 79.83 (SD ± 13.82). There were also statistically significant correlations between attitude and self-efficacy, and labor support behaviors and its dimensions. Multiple linear regression analysis results indicated that interests in occupation, attitude, and self-efficacy were predictors of labor supportive behaviors.

**Conclusion:**

Midwives’ level of interest in the profession, attitude, and self-efficacy of labor support were significantly associated with labor support behaviors. Thus midwives’ interest in their profession, along with their attitudes and self-efficacy could usefully be developed to enhance their supportive behaviors during labor.

**Supplementary Information:**

The online version contains supplementary material available at 10.1186/s12912-023-01197-w.

## Introduction

The words "labor support" refer to supporting behaviors and ongoing non-pharmacological care to women during the labor process [1]. Such support includes physical touch, massage, showers, cold or warm compressions, continual follow-up, reassurance, encouragement, informational support, and non-pharmacologic recommendations [1, 2]. Particularly in the hospital setting, support should be provided by doulas or midwives due to the separation from the more familiar home environment [2, 3]. Yet in some low-income countries including Iran, a spouse may not be permitted in the birthing room and as such, support by the midwife becomes more important during labor and childbirth [4].

Outcomes related to increased support during labor in primiparous women can include increased labor progression, along with reduced rates of cesarean section, use of ephedrine anesthetic, and synthetic oxytocin [4–6]. In contrast, poor support during labor and birth can result in increased postpartum mental and psychological ill health, depression, post-traumatic stress disorder, and poorer experiences [7, 4]. Nevertheless, studies of this nature remain largely absent in the context of Iran, where birthing rooms remain crowded with little or no labor support provided [8].

There are many factors, both individual and organizational which influence the provision of labor support behavior by midwives. For example, in a study by Elmashad et al. (2018), the facilitation of evidence-based decision making and effective pain management were identified as effective factors in labor support [9]. In another study, demographic characteristics such as age, education and work experience of midwives and in other studies have also emphasized the authority of midwives in providing labor support effectively [1, 10–12]. Therefore, it will be important to examine a variety of midwives characteristics in relation to the provision of labor support. In addition, the workplace cultures apparent in the birthing environment [12], and relationships with physicians [10–13] have also been found to influence the effectiveness of profession labor support (PLS). For example, birth workers have frequently reported how certain behaviors of physicians (e.g., offering epidural analgesia and augmenting and monitoring labor) have limited their ability to provide the most effective care and support in labor [10, 12, 13]. Therefore, midwives’ clinical environment will also be an important characteristic to consider in examining labor support. Lack of awareness in relation to the benefits of labor support and poor attitudes toward the provision of labor support by midwives may also affect the supportive behavior of midwives [2, 14]. Furthermore, subjective norms such as the provision of supportive behaviors being valued from the point of view of others [15], self-efficacy [14, 16], midwives' approaches to performing labor support [14–16] could also influence the effectiveness of any labor support given. Nevertheless, as few studies of this nature are conducted in Iran, it is not yet known which factors may affect the provision of labor support for those birthing in Iran.

According to Social Cognitive Theory, one’s behavior is understood to be determined by four factors: goals, outcome expectancies, self-efficacy, and socio-structural variables [17]. Attitudes and self-efficacy are both also individual factors of particular importance in influencing the provision of support and can improve midwives’ supportive behaviors [16]. As midwifery care is centered around the giving of support, and the midwifery profession itself requires self-efficacy in order to provide support during labor, midwives who have higher levels of self-efficacy are known to provide more effective and frequent support [18]. Indeed, moderate levels of supportive behaviors (M = 3.04, SD = 0.33, range = 0–5), positive relationships with attitude, self-efficacy, and work experience have been found to be significantly related to professional labor support behaviors elsewhere [16]. One’s own personal birth experience can also be positively correlated with attitudes about and intent to provide PLS [19], along with age and length of experience [1, 12]. Furthermore, good knowledge in relation to pain relief in labor and working at private primary hospitals have also been found to be statistically significant in relation to favorable attitudes and clinical practice [20, 21]. Other factors associated with the low levels of supportive behavior provided by midwives for women in labor relate to their workload and shift length [14, 18]. Significantly, the results of some Iranian studies have identified dissatisfaction in relation to the informational and emotional support received during labor and after childbirth [22]. However, other studies indicated a good level of support during labor [23]. This suggests a level of incongruence within the literature on this issue and identified a need for further study in this area. The care model provided in Iran is different from other countries. Although midwifery has been defined as an independent profession in Iran [24], no independent professional mechanisms have been considered for it. Therefore, in the healthcare system, perinatal care is predominantly isolated and managed with a biomedical approach. Indeed, perinatal care is often provided by midwives, yet it is often led by gynaecologists and/or obstetricians [25]. This can lead to the unnecessary medicalization of childbirth and inhibit midwives’ role in providing support. In most Iranian hospitals, and particularly government hospitals, spouses are not permitted to attend births. Instead, several female relatives are simultaneously present due to cultural and religious restrictions. Since the midwife is required to provide effective labor support in this context, it will be important to identify how support during labor is provided and it’s affecting factors from midwives’ perspective. Considering the above, the aims of the current study were to determine the attitudes, self-efficacy during labor support and supportive behaviors from the perspectives of midwives employed in hospitals in an urban area of Iran and to explore the relationship between participants’ socio-demographic characteristics, attitude and self-efficacy during labor support and supportive behaviors from the perspectives of midwives employed in hospitals in an urban area of Iran.

### Theoretical framework

We applied the Social Cognitive Theory of Bandura (1986), and evidence from the literature as a conceptual framework for this study. Bandura’s theory addresses the dynamic relationship between individuals, the environment, and behavior. The view of the two-sided determinism theory creates personal factors in the form of cognitive, affective and biological behavior, and behavior and environmental effects that lead to a mutual interaction. The concept of work experience in health care was also included, as proposed by the literature [1, 16, 17].

Self-efficacy refers to beliefs about an individual's ability to perform a particular task or action. Experience allows the midwife to understand what is happening and how to react. This trust or belief in one’s capabilities represents optimal self-efficacy as a component of the Social Cognitive Theory [16, 26, 27]. According to Bandura (1986), the behavior of individuals is influenced by what they think or believe, and how they feel. To facilitate the protection of supportive workers, it will be essential to understand their attitudes toward support work. Ultimately, the conceptual theory drawn upon within the present study is influenced by social cognitive theory, and relates to a dynamic relationship between individuals, their environment, and behavior [17].

## Methods

### Participants and settings

This was a cross-sectional study involving midwives employed in 15 labor wards of 15 governmental (referral or non-referral) hospitals in an urban area of Tehran Province, Iran. Hospital sites were selected using convenience sampling. Due to limitations in access to hospitals, it was not possible to randomly select them. The sample size was calculated to be 200 according to the formula, taking into consideration the 95% confidence level, an 80% power calculation, and by assuming a correlation of 0.2 between attitude and self-efficacy of labor support and supportive behaviors in labor.

$$\begin{array}{cc}w=\frac{1}{2}\mathrm{ln }\frac{1+r}{1-r} & w=\frac{1}{2}\mathrm{ln}\frac{1+0.2}{1-0.2}=0.201\end{array}$$

$$\begin{array}{cc}n=\frac{\left(z_{1-a/2}+z_{1-\beta}\right)^2}{w^2}+3&n=\frac{\left(1.96+0.84\right)^2}{\left(0.201\right)^2}\end{array}+3=200$$

Convenience sampling was used to recruit participants from December 2016 to September 2017 until the required sample size was reached. In this process, the primary researcher invited the midwives working in the 15 labor wards who met the inclusion criteria to complete the questionnaires once participant information and informed consent had been given. Inclusion criteria comprised at least six months of work experience in labor ward. Participants who did not complete over 90% of responses were excluded from the study.

### Data collection and instruments

The data collection tools consisted of a personal characteristics tool, the attitudes toward Labor Support Questionnaire, the Self-efficacy Labor Support Scale, and the Labor Support Questionnaire. The data was collected by a single researcher (K.H) and participants completed the entire questionnaire just once.

The attitudes toward Labor Support Questionnaire consisted of four questions, with answers recorded via a seven-point Likert scale. Scores for this tool range from 7 to 28 where a lower score is indicative of a more negative attitude and higher score is indicative of a more positive attitude [14]. The Self-efficacy Labor Support Scale consisted of 14 questions, with answers again recorded via a seven-option Likert scale with scores ranging from 14 to 98, indicating either negative or positive self-efficacy, respectively [18]. The Labor Support Questionnaire contained 27 items, with answers recorded again via a six-option Likert with score classifications of weak (0–44), middle (45–89), and good (90–135) [28, 29].

After obtaining consent from the designers, the tools were considered for backward-forward translation and cultural adaptation. The translation of the scales from the original language to Farsi was done by two professors familiar with English and medical texts. Back translation was also done with the help of two other people with the same characteristics. Face validity was undertaken by ten midwives following translation. To check the content validity, ten experts proficient in the subject of research and instrument design were requested to provide their corrective views on the scale.

In order to confirm the construct validity, confirmatory factor analysis was performed. In order to perform confirmatory factor analysis, sampling was done on qualified midwives (*n* = 213). The fit indices examined in both tools include χ2 /df less than 5, Root Mean Square Error of Approximation (RMSEA) less than 0.08, as well as goodness of fit index (GFI), comparative fit index (CFI), adjusted goodness of fit index (AGFI), normalized fit index (NFI) and non-normalized fit index (NNFI) greater than 0.9 were considered favorable [30]. Based on this model, standardized factor loadings above 0.3 are considered moderate to strong factor loading [31]. In general, all the model fit indices provided in tools had good fit and confirmed the assumed model in tools.

In order to confirm the reliability of the tools, internal consistency and temporal stability was measured. The internal consistency of the Self-efficacy Labor Support Scale, the Labor Support Questionnaire, and attitude toward labor support was confirmed with Cronbach's alpha of 0.93, 0.96, and 0.90, respectively, by sampling 30 midwives. To check the time stability of the scales, the test–retest approach was used. Midwives (*n* = 30) with at least six months experience of providing labor care were selected. Each person completed the tools two weeks apart. The temporal stability of tools was confirmed with the Spearman coefficient of 0.72, 0.71, and 0.69. More details about each of the tools are given in the related published articles [32, 33].

### Data analysis

Data analysis was performed using SPSS software v. 21. Descriptive and inferential statistics were used to make sense of the data collected. To compare the attitude and self-efficacy of labor support behaviors (quantitative variables) among personal characteristics variables (categorical variables), an independent t- test and ANOVA were used. Pearson’s correlation coefficient test was used to determine the relationship between the attitude and self-efficacy of labor support behaviors with personal characteristics variables that were considered quantitative variables.

In order to assess the effect of each independent variable on labor support behavior, we entered all variables that had a *p*-value < 0.05 into a multiple linear regression analysis by using a backward strategy. Regression assumptions which included normal data distribution, homogeneity of residue changes, linear distribution of outliers were investigated prior to assessment of multivariate analysis. Differences were considered significant at *P* < 0.05, whereas differences between 0.05–0.10 were considered to be close to significant.

### Ethical considerations

The Ethics Committee of Iran University of Medical Sciences (IR.IUMS.FMD.1396.9511373005), Tehran, Iran approved current study protocol. Written informed consent was obtained from the participants after they were completely informed of the study purpose and procedures. All participants were assured of the confidentiality of their information. The questionnaires were completed by the participants.

## Results

A total of 227 midwives who met the inclusion criteria agreed to participate in current study, 213 of whom completed the questionnaire for an effective response rate of 93.83% (213/227) (Supplementary Table 1). The mean age of participants was 32.64 (SD ± 9.4) years. Most participants (77.5%) had been educated in governmental university. The education level of 87.3% (n = 186) participants measured to be Bachelor of Science (BSc level). Over half, 57.5% (*n* = 123) of participants had less than 5 years of work experience in labor wards (Table 1). Results presented in Table 2 demonstrate that the attitude toward support in labor was 94.8% higher than the median value of 16. The midwives' self-efficacy labor support was 96.2% above the median value of 49. In relation to labor support behavior, 73.7% of the midwives had a good level of supportive behaviors (90–135). The mean score of supportive behaviors was 100.99 and 95.8% of midwives had labor support behaviors above the median value of 67.

**Table 1:** Participants’ socio-demographic characteristics

Personal Characteristics	Number (%)
Age (Year)	
≤ 29	106 (49.8)
30 – 39	57 (26.7)
40 – 49	34 (16)
≥ 50	16 (7.5)
Type of university of education	
Governmental university	165 (77.5)
Non-governmental university	48 (22.5)
Education level	
Associate's degree	7 (3.3)
Bachelor of Science	186 (87.3)
Master of Science	20 (9.4)
Work experience in labor ward	
< 5	123 (57.7)
5 – 10	39 (18.3)
10 – 15	12 (5.7)
15 – 20	10 (4.7)
> 20	29 (13.6)
Interest in the profession	
Very much	68 (31.9)
Much	84 (39.4)
Medium	49 (23)
Little	5 (2.4)
Very little	7 (3.3)
Marital status	
Single	90 (42.2)
Married	118 (55.4)
Divorced and widowed	5 (2.4)
Type of hospital	
Educational hospitals	102 (47.9)
Non-educational hospitals	111 (52.1)

**Table 2:** Attitude and self-efficacy of labor support

Variable	Median	Number (%)	Mean	Std. deviation	Minimum	Maximum	Median
Attitude toward labor support (Range: 4–28)	Median and lower (4–16)	11(5.2)	24.79	4.14	5	28	16
	Above the Median (17–28)	202 (94.8)					
Self-efficacy of labor support (Range: 14–98)	Median and lower (14–49)	8 (3.8)	79.83	13.82	37	98	49
	Above the Median (50–98)	205 (96.2)					
Labor Support Behavior (Range: 0–135)	Median and lower (0–67)	9 (4.2)	100.99	18.08	34	135	67

**Fig. 1:**
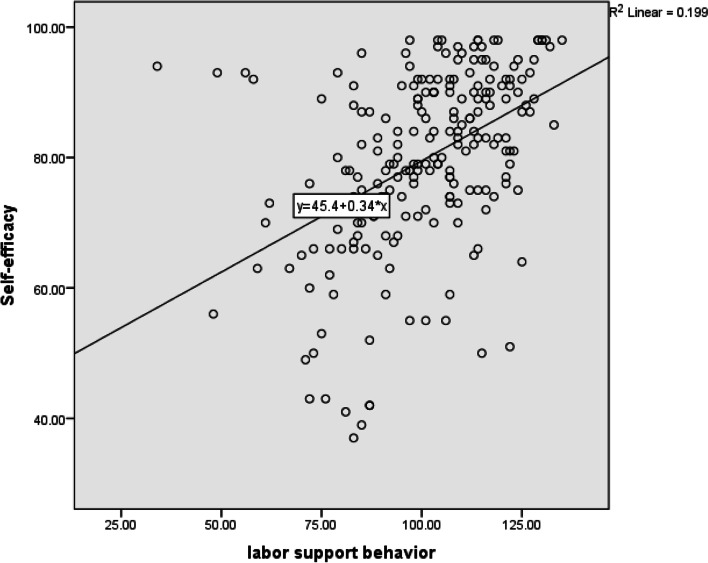
Correlation between self-efficacy of labor support and labor support behavior

**Fig. 2:**
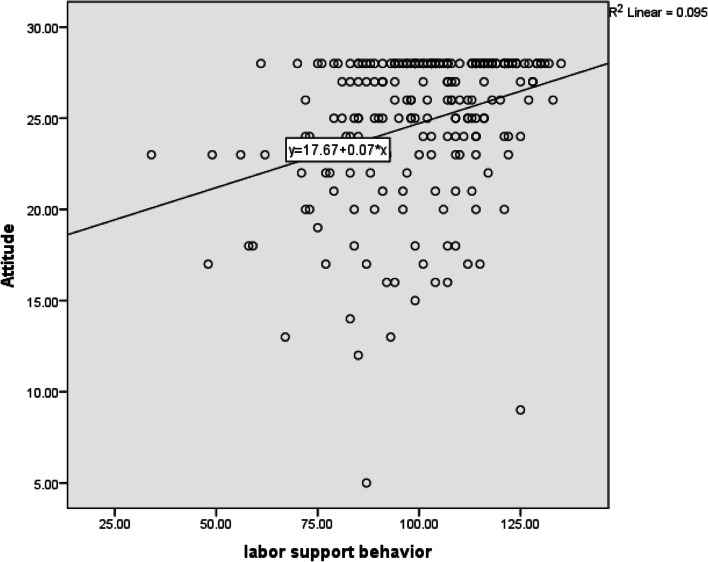
Correlation between attitude and labor support behavior

In terms of supportive behaviors during labor and their dimensions, the midwives reported labor support behaviors of 74.80 (SD ± 13.39). The highest scores related to the informational support dimension (Mean = 79.62 and SD = 17.13) and the lowest for the tangible support dimension (Mean = 65.88 and SD = 16.69). There were statistically significant correlations between attitude and self-efficacy, and labor support behaviors and its dimensions (Table 3) (Fig. 1 and 2).

**Table 3:** The correlation of labor support behaviors, attitude and self-efficacy

Dimensions of LSB^a^	^b^Mean	^€^SD	Min	Max	Attitude		Self-efficacy	
					^**$**^***r***	*p	^**$**^***r***	*p
Informational support	79.64	17.13	20	100	0.21	0.002	0.29	< 0.001
Tangible support	65.88	16.69	23.33	100	0.35	< 0.001	0.43	< 0.001
Advocacy	75.14	15.23	16	100	0.15	0.02	0.40	< 0.001
Emotional	77.6	13.88	24.62	100	0.31	< 0.001	0.40	< 0.001
Total	74.8	13.39	25.19	100	0.3	< 0.001	0.44	< 0.001

^a^Labor Support Behavior

^b^The scores are based on the 100 (Range of scores 0–100. with 0 = Weak, 100 = well)

^*^P value < 0.05 was considered significant and ^$^r is Pearson correlation coefficient

Age, education level, work experience, length of work experience in a labor ward, type of hospital, and marital status had no statistically significant relationships with labor support behaviors. Conversely, the type of university attended (p = 0.038), and interest in the profession (*p* = 0.004) had statistically significant relationships with labor support behaviors (Table 4). We introduced these four variables (type of university during education, interest in the profession, attitude, and self-efficacy) into a multiple regression model analysis using a backward strategy. Of these four variables, the degree of interest in the profession, attitude, and self-efficacy remained in the model. Participants who had medium and much interest in the profession scored 7.42 and 7.005 fewer units in relation to the labor support behaviors than participants who had much interest in the profession. For one score increase in the attitude and self-efficacy, the score of the labor support behaviors increased by 0.50 and 0.47 units, respectively. Accordingly, 23% of changes in labor support behaviors were justified by the degree of interest in the profession, attitude, and self-efficacy (Table 5).

**Table 4:** The relationship of midwives' individual characteristics with the labor support behaviors

	n (%)	Mean	SD	*P* value
Age (Year)				
≤ 29	106 (49.8)	100.3	18.9	0.75
30 – 39	57 (26.7)	102	18.22	
40 – 49	34 (16)	100.02	16.80	
≥ 50	16 (7.5)	103.93	15.62	
Type of university during education				
Governmental university	165 (77.5)	99.6	17.8	**0.038**
Non-governmental university	48 (22.5)	105.75	18.44	
Education level				
Associate's degree and Bachelor of Science	193 (90.6)	102.21	18.64	0.52
Master of Science	20 (9.4)	98.5	14.05	
Work experience in labor ward (Year)				
< 5	123 (57.7)	100.51	19.3	0.95
5 – 10	39 (18.3)	102.33	15.35	
10 – 15	12 (5.7)	100.41	20.7	
15 – 20	10 (4.7)	104.1	18.16	
> 20	29 (13.6)	100.4	15.85	
Interest in the profession				
Very much	68 (31.9)	107.5	16.22	**0.004**
Much	84 (39.4)	98.73	18.4	
Medium	49 (23)	96.57	16.73	
Little and very little	12 (5.7)	98	23.18	
Marital status				
Single	90 (42.2)	100.27	18.8	0.702
Married	118 (55.4)	101.25	17.76	
Divorced and Widowed	5 (2.4)	100.14	17.8	
Type of hospital				
Educational hospitals	102 (47.9)	100.44	19.2	0.67
Non-educational hospitals	111 (52.1)	101.5	17.1

**Table 5:** Multiple linear regression analysis

**Variable**	**B**	**Beta (**^a^**CI 95%)**	**t**	^**‡**^***P*** **value**
Interest in the profession				
Very much (reference)	0	-	-	-
Much	-7.005	- 0.19 (-12.13 up to -1.87)	-2.69	**0.01**
Medium	-7.42	-0.17 (-13.35 up to -1.5)	-2.47	**0.01**
Little and very little	-7.73	-0.09 (-17.56 up to 2.09)	-1.55	0.12
Type of university during education				
Non-governmental university	0	-	-	-
Governmental university	-3.60	-0.08 (-8.79 up to 1.57)	-1.37	0.2
Attitude	0.50	0.11 (-0.08 up to 1.08)	1.69	0.1
Self-efficacy	0.47	0.36 (0.30 up to 0.65)	5.39	**0.001**

^‡^*P* value < 0.05 and *P* value = 0.05–0.10 were considered significant and close to significant respectively

^a^Confidence Interval, Adjusted R Square = 0.23, R Square = 0.25, R = 0.50

## Discussion

The current study examined the attitudes, self-efficacy during labor support and supportive behaviors from the perspectives of midwives employed in hospitals in an urban area of Iran. It also explored the relationship between their socio-demographic characteristics, attitude and self-efficacy during labor support and perceived supportive behaviors. In the present study, the mean score of support behaviors was 74.80 (SD ± 13.39). The dimensions of labor support behaviors indicated that informational support and tangible support had the highest and lowest mean scores, respectively. Interest in the profession, attitude, and self-efficacy of labor support were predictors of labor support behaviors in Iranian midwives.

Regarding the self-efficacy score of labor support, the findings of the research showed that the self-efficacy of labor support was 96.2% higher than the average (49). The mean and standard deviation of the self-efficacy scores for labor support were 79.83 and 13.82 respectively, which indicated high self-efficacy in participants. To compare these results with existing literature, the mean and standard deviation of the self-efficacy of labor support in the study of HOA (2015) were 63.65 and 11.49 respectively, indicating the average self-efficacy of nurses regarding labor support [16]. In another study, the mean score of self-efficacy of labor support was reported as 30.28, which was close to the mean score of the tool [30, 34]. These results did not match the findings of the present study. Such differences could be due to the fact that in Iran, midwives are required to undergo a training course on providing effective support during labor, which is known to improve self-efficacy in midwives [35].

In relation to determining the score of labor supportive behaviors in midwives, the average overall score of labor supportive behavior was 74.80 (range 0–100). The most support was in the field of information and the least support was related to the tangible dimension. In another study, where the measurement tool was similar to the current research tool, the average overall score of the supportive behavior of labor was 3.04 (range 0–5) [16]. The results of these two studies are consistent. Similarly, the emotional dimension had the highest score, and tangible support had the lowest score, which was consistent with the findings of our research in that the lowest score was related to the tangible dimension. Moreover, the mean and standard deviation of the score of the labor supportive behavior elsewhere was found to be 4.54 and 0.40 (range 0–5), respectively [36]. The results of the present study were consistent with the findings of this research in terms of the high overall score of labor support behavior. Yet in terms of dimensions, the results of this study did not match our own, as the information dimension had the highest score, and the tangible dimension had the lowest score. Such inconsistencies may be due to Iranian midwives’ excessive responsibilities and workloads, which can prevent the provision of effective support [37, 38]. Similarly, different cultural conditions governing the societies, the number of assessed samples and the dispersion of the sampling centers may also explain inconsistencies in this area. In HOA's (2015) study, the tangible support dimensions scored lower [16], in line with the results of the current study and indicative of a lower mean score for the tangible support dimension. The reason for this low score is suggested to be due to the use of advanced technologies (monitoring of the fetal heart, use of oxytocin, etc.) before epidural anesthesia [14, 16]. Other differences in findings may be due to the use of different measurement tools and sampling [23]. Yet other studies highlight a congruence of findings in line with ours [14, 15, 36].

The results presented here are not surprising given that individuals’ behavior is affected by their emotional state and/or significant events, beliefs and anticipated consequences [39]. In this sense our findings contribute to understandings in relation to the Social Cognitive Theory [17]. To change poor labor support practices, beliefs regarding the benefits of supportive care must be changed [18, 40–42]. As such, evidence-based promotion of the benefits of effective support during labor may influence attitudes and in turn, promote improved practice in this area.

In determining the relationship between labor support self-efficacy and labor support behaviors, our findings demonstrated a positive and significant correlation between these two variables, consistent with the findings of [18, 36]. This relationship was again congruent with our theoretical perspective, which suggests that individuals with high sense of self-efficacy will enhances strong commitment to specific behavior [17]. This study’s finding was also coincides with those of previous studies identifying a positive relationship between self-efficacy and health-promoting behavior [43], along with self-efficacy and the application of evidence- based practice [44, 45].

Our findings are consistent with those of Vasegh Rahimparvar et al. (2012) regarding the absence of a statistically significant relationship between labor support behaviors and the personal characteristics of the students and midwives under study [46]. Similarly, our findings were consistent with the results of HOA (2015) in that no significant relationship was identified between education and labor support behaviors elsewhere. While in the same study, there was a weak relationship between work experience and labor support behaviors (*r* = 0.28) [16]. In another study conducted in the United States of America, only age (*r* = 0.39) and experience in providing care (*r* = 0.31) were found to be related [1], though the age of these participants was higher than the ages of current participants. While in the present study, the average age and work experience of the midwives participating in the study were 32.64 and 6.91 years, respectively. Such differences point to a need for larger and more generalizable studies in this area.

Regarding the predicting variables of labor support behaviors, among the variables related to individual characteristics that were included in the model, only the variables of interest in the profession, attitude and self-efficacy of labor support remained in the model. Accordingly, 23.5% of changes in the dependent variable of labor support behaviors were justified by the independent variables of interest in midwifery profession, attitude and self-efficacy of labor support that they were intrapersonal factors and the effect of related interpersonal and environmental-organizational factors on the occurrence of labor support behavior was not investigated. Our findings were consistent with the results of HOA (2015) regarding the stronger relationship of self-efficacy of labor support with supportive behaviors from the perspective of midwives compared to attitude. The intensity of correlation for self-efficacy compared to attitude was also measured to be higher [16], and consistent with the findings of the present study. Such findings highlight the need for increased self-efficacy in midwifery populations in pursuit of improved midwifery support during labor.

### Strength and limitation

A key strength of current study is that it has included participants from referral and non-referral hospitals and has reported the results based on the regression model to predict the labor support behaviors according to personal related factors. Our sample of midwives was recruited from 15 government hospitals in Tehran Province, Iran. As such, our sample is not representative of all midwives in Iran. Consequently, these findings cannot be generalized to other areas and in particular, private hospitals. Due to the limited access to hospitals and midwives working in each hospital, random sampling was not possible. Equally, as our data collection ended five years ago, more recent data is also required in pursuit of comparative work along with deeper and more contemporary understandings. Further efficient studies on the relationship between attitude and self-efficacy of labor support with supportive behaviors among midwives in private hospitals and other Iranian provinces should be conducted in future with larger and more representative samples. In the current study, we assessed only the personal factors, attitudes, and self-efficacy of labor support. Future studies could usefully examine other influencing factors such as intrapartum midwives’ knowledge of labor support and current best evidence, organizational characteristics, and management support with labor support behaviors and their dimensions. The tangible support dimension had the lowest score; therefore, we suggest that additional studies should be conducted that address this dimension in particular.

## Conclusions and implications

The results of the current study revealed good scores in relation to labor support behavior by midwives. Tangible and informational support dimensions of labor support behavior had the lowest and the highest scores, respectively. The levels of interest in the profession, attitude, and self-efficacy of labor support were significantly associated with labor support behaviors. Thus, health care managers and policymakers could usefully take measures to improve labor support behaviors of midwives. This may include promotion of the midwifery profession as a valuable entity. Policymakers could also usefully be made aware of the labor support behaviors of midwives and focus on interventions that target increased interest in the profession, attitude, and self-efficacy of labor support. Our findings provide evidence for the use of labor support behaviors in routine care during labor. Yet ultimately, more emphasis is required upon the dimension of tangible support.

## Supplementary Information

**Additional file 1: Supplementary Table 1.** Frequency distribution of midwives in selected hospitals in Tehran, Iran, during 2016-2017.

## Data Availability

The data that support the findings of current study are available from [Leila Amiri-Farahani] but restrictions apply to the availability of these data, which were used under license for the current study, and so are not publicly available.
