# Supplementary material for: The relationship between attitude and self-efficacy of labor support with supportive behaviors from the perspective of midwives
Source: BMC Nurs. 2023 Feb 7;22:36. doi: 10.1186/s12912-023-01197-w (PMC9903519; doi:10.1186/s12912-023-01197-w)
Supplement: Supplementary file 1 — Additional file 1: Supplementary Table 1. Frequency distribution of midwives in selected hospitals in Tehran, Iran, during 2016-2017. [file 12912_2023_1197_MOESM1_ESM.docx]

**Supplementary Table 1: Frequency distribution of midwives in selected hospitals in Tehran, Iran, during 2016-2017**

| **Hospitals** | **n (%)** | **Total number of midwives in labor ward** | **Type of hospital** |
| --- | --- | --- | --- |
| Shahid Akbar Abadi | 30 (14.1) | 46 | Governmental/ referral |
| Mahdiyeh | 17 (8) | 35 | Governmental/ referral |
| Ziaian | 25 (11.7) | 28 | Governmental/ non-referral |
| Baharlu | 19 (8.9) | 23 | Governmental/ non-referral |
| Fatemeh Al-Zahra | 15 (7) | 19 | Governmental/ non-referral |
| Firouzabadi | 14 (6.6) | 22 | Governmental/ non-referral |
| Lolagar | 9 (4.2) | 14 | Governmental/ non-referral |
| Imam Hossein | 10 (4.7) | 20 | Governmental/ referral |
| Yaft Abad | 12 (5.6) | 15 | Governmental/ non-referral |
| Emam Sajjad | 17 (8) | 21 | Governmental/ non-referral |
| Arash | 24 (11.3) | 38 | Governmental/ referral |
| Shohadaye Tajrish | 4 (1.9) | 11 | Governmental/ referral |
| Yas | 6 (2.8) | 13 | Governmental/ referral |
| Firoozgar | 5 (2.4) | 12 | Governmental/ non-referral |
| Rasoul Akram | 6 (2.8) | 14 | Governmental/ referral |
| Total | 213 (100) | 331 | - |
